# Work-family conflict and burnout among Chinese female nurses: the mediating effect of psychological capital

**DOI:** 10.1186/1471-2458-12-915

**Published:** 2012-10-29

**Authors:** Yang Wang, Ying Chang, Jialiang Fu, Lie Wang

**Affiliations:** 1 Department of Social Medicine, School of Public Health, China Medical University, No. 92 Beier Road, Heping District, Shenyang 110001, Liaoning, People’s Republic of China

**Keywords:** Burnout, Work-family conflict, Psychological capital

## Abstract

**Background:**

Burnout among nurses not only threatens their own health, but also that of their patients. Exploring risk factors of nurse’ burnout is important to improve nurses’ health and to increase the quality of health care services. This study aims to explore the relationship between work-family conflict and burnout among Chinese female nurses and the mediating role of psychological capital in this relationship.

**Methods:**

This cross-sectional study was performed during the period of September and October 2010. A questionnaire that consisted of the Maslach Burnout Inventory-General Survey (MBI-GS), the work-family conflict scale and the psychological capital questionnaire (PCQ-24) scale, as well as demographic and working factors, was distributed to nurses in Liaoning province, China. A total of 1,332 individuals (effective response rate: 78.35%) became our subjects. Hierarchical linear regression analyses were performed to explore the mediating role of psychological capital.

**Results:**

Both work interfering family conflict and family interfering work conflict were positively related with emotional exhaustion and cynicism. However, work interfering family conflict was positively related with professional efficacy whereas family interfering work conflict was negatively related with it. Psychological capital partially mediated the relationship of work interfering family conflict with emotional exhaustion and cynicism; and partially mediated the relationship of family interfering work conflict with emotional exhaustion, cynicism and professional efficacy.

**Conclusion:**

Work-family conflict had effects on burnout and psychological capital was a mediator in this relationship among Chinese nurses. Psychological capital was a positive resource for fighting against nurses’ burnout.

## Background

Burnout is defined as a syndrome of emotional exhaustion, cynicism and reduced professional efficacy [1] that occurs among various people-oriented professions such as health care and social services [2]. Burnout is reported to be associated with many symptoms and possible consequences such as depression, absenteeism, substance abuse [3]. Nurses are considered a risk population with high levels of burnout [4,5]. Burnout among nurses threatens not only their own health, but also that of their patients. For example, Halbesleben et al [6] reported that burnout of nurses was associated with the perception of lower patient safety. Huge population basis and the severe nursing shortage in China make Chinese nurses more likely to experience burnout. In China, the ratio of nurses to general population is 1:1750, which is considerably lower than that in developed countries (1:140–1:320) [7]. Therefore, exploring risk factors of nurse’ burnout is important to improve nurses’ health and to increase the quality of health care services in China.

Work-family conflict has been found to be positively associated with burnout in previous studies [8,9]. Work and family are two important domains of adult life. However, the role expectations of these two domains are always incompatible that participation in one domain makes it difficult to participate in the other one. Work-family conflict is a bi-directional conflict including both work interfering family conflict (WIF) and family interfering work conflict (FIW) [8,10]. In china, dual-career families are common, both males and females need to take household responsibilities and tasks. Compared to other occupational populations, medical staffs are devoting much time and energy to work and experiencing higher level of work-family conflict in China [11]. However, though the detrimental effect of work-family on burnout has been tested in other occupational populations [10,12], few studies have been done among nurses to our knowledge.

With an increasing recognition of the value of positive organizational behavior, organizations sought to improve employees’ physical and psychological health by strengthening the psychological resources of employees. Psychological capital (PsyCap) is an important concept drawn from positive organizational behavior [13] and has been reported as a positive resource for improving employees’ job performance [14], job satisfaction [15], employees’ well-being over time [16], and for combating employees’ stress and turnover [13]. Self-efficacy, hope, optimism and resilience, which can be measured, developed, and effectively managed, are identified as the components of PsyCap [17-19]. In addition, PsyCap was identified as a mediator in the relationship between supportive organizational climate and employees’ performance [19] and also a mediator in the relationship between job burnout and turnover intention among Chinese nurses [20]. Although both the association between work-family conflict and burnout and the association between PsyCap and burnout have been investigated in previous studies [20,21], PsyCap as a mediator in the relationship between work-family conflict and burnout has not been confirmed yet to our knowledge. Theoretically, PsyCap is state-like and open to change, therefore, PsyCap of nurses who experience high level of work-family conflict might decrease over a prolonged period, and further result in high level of job burnout of nurses.

The aim of the present study is twofold. First, we will examine the relationship between work-family conflict (WIF and FIW) and burnout among Chinese nurses. Second, we will examine the mediating role of PsyCap in the relationship between work-family conflict and burnout or, in other words, whether work-to-family conflict affects burnout to some extent via psychological capital. If this is the case, we could not only look for interventions to decrease work-family conflict of nurses, which are relative difficult to realize in China, but also focus on development and investment in nurses’ PsyCap. Therefore, it is important to understand the mediating role of PsyCap in this relationship to prevent and treat burnout adequately.

## Method

### Study design and sample

A cross-sectional study was conducted in Liaoning province of China during September and October 2010. Based on the geographic division and economic development of Liaoning province, the whole province was divided into five regions. One city in each region was randomly selected. One large general hospital (>500beds) was randomly selected in each sampled city, and if the sampled city was a megalopolis, one more large general hospital was randomly selected. A total of six large general hospitals were selected eventually. Because male nurses only took account of <1% of Chinese nurses [22], we only focused on female nurses in this study. Half of female nurses were randomly selected from each sampled hospital. Eventually, 1,700 female nurses were recruited in this study. A pool of 1,332 female nurses (effective response rate: 78.35%) from 6 selected hospitals constituted the potential study sample. After obtaining the informed consent to conduct this survey, a self-administered questionnaire was distributed to recruited registered nurses.

This study received ethics approval from the Committee on Human Experimentation of China Medical University.

### Measurement of burnout

Burnout was measured with the Maslach Burnout Inventory- General Survey (MBI-GS), developed by Maslach and Jackson [23,24]. The MBI-GS consisted of 15 items and three dimensions: emotional exhaustion, cynicism and professional efficacy. Emotional exhaustion dimension was measured by five items, cynicism dimension was measured by four items, and professional efficacy dimension was measured by six items. All the items were scored on a Likert scale from 0 (never) to 6 (every day). Higher scores on emotional exhaustion and cynicism dimensions and lower scores on professional efficacy dimension indicated higher levels of burnout.

The Chinese version of the MBI has been used widely in Chinese population and demonstrated satisfactory reliability and validity [4,5]. In the present study, the Cronbach’s alpha for the total scale was 0.883. Cronbach’s alpha coefficients of emotional exhaustion, cynicism, and professional efficacy were 0.942, 0.939, and 0.916, respectively.

### Measurement of work-family conflict

Work-family conflict was measured by two subscales: WIF scale and FIW scale [8]. The WIF scale measured the extent to which work demands interfere with family-related obligations, while FIW scale measured the extent to which family demands interfere with work-related obligations [8]. The total scale consisted of 18 items and each of the two subscales was measured by nine items. All the items were scored on a 5-point Likert scale from 0 (strongly disagree) to 5 (strongly agree). Responses for each of the two subscales were summed and averaged to get an average score for WIF and FIW, respectively. Higher values of subscales indicated higher levels of WIF or FIW. The Chinese version of the work-family conflict scale has been demonstrated good reliability and validity [25,26]. In the present study, the Cronbach’s alpha for the total scale was 0.900. Cronbach’s alpha coefficients of WIF and FIW were 0.864 and 0.900, respectively.

### Measurement of psychological capital

PsyCap was measured with the PCQ-24 questionnaire which was developed by Luthans et al [27]. The PCQ-24 questionnaire consisted of 24 items and four dimensions: self-efficacy, hope, resilience and optimism. Each of the four dimensions was measured by six items. All the items were scored on a Likert scale from 1 (strongly disagree) to 6 (strongly agree). Higher values indicated higher levels of experienced psychological capital.

The Chinese version of the PCQ-24 has been used in Chinese studies and has demonstrated satisfactory reliability and validity [28,29]. In this study, we summed up responses for 24 questions and averaged to get an average score as the indicator for total PsyCap. Cronbach’s alpha coefficient of the total scale was 0.928. Cronbach’s alpha coefficients of self-efficacy, hope, resilience and optimism were 0.853, 0.862, 0.816 and 0.786, respectively.

### Demographic and working characteristics

Age, educational level, marital status, monthly income and weekly working hours were obtained in this study. ‘Education’ was categorized as ‘High school or under’, ‘Junior college’, and ‘Undergraduate or above’. ‘Marital status’ was categorized as ‘Single’, ‘Married/Cohabitation’, and ‘Divorced/Separated/Widow’. ‘Monthly income’ was categorized as ‘≤3000RMB (≈470 dollars)’ and ‘>3000RMB’. ‘Weekly working hours’ was categorized as ‘≤40 hours’ and ‘>40 hours’.

### Statistical analysis

All analyses were performed using the SPSS 17.0 program and all statistical tests were two-sided (*α* = 0.05). Distributions of dimensions of burnout in categorical demographic characteristics were tested by one-way ANOVA. Pearson correlation was performed for testing the relationship between work-family conflict and burnout. Baron and Kenny’s [30] technique was used for testing the mediating effect of PsyCap in the relationship between work-family conflict and burnout. According to Baron and Kenny [30], the following conditions should be satisfied for establishing mediation: (1) The independent variable (WIF/FIW)is significantly associated with the dependent variable (Emotional exhaustion/Cynicism/Professional efficacy); (2) The independent variable (WIF/FIW) is significantly associated with the mediator (PsyCap); (3) The mediator (PyCap)is significantly associated with the dependent variable (Emotional exhaustion/Cynicism/Professional efficacy) , and the effect of the independent variable (WIF/FIW) on the dependent variable (Emotional exhaustion/Cynicism/Professional efficacy) shrinks upon the addition of the mediator (PyCap)to the model (partial mediator). If the independent variable does not affect the dependent variable when the mediator is added to the model, then full mediation is established.

All the continuous variables were centralized in order to avoid multicollinearity [31] before regression analyses were performed. Moreover, tolerance and variance inflation factor were used to check for multicollinearity. We performed Pearson correlation and one hierarchical linear regression analysis for each of the three burnout dimensions to test the mediating effect. In step 1 of the hierarchical linear regression analyses, the control variables were put in the model. In the present study, we keep all age, marital status, educational level, monthly income and weekly working hours in the model as potential confounders. Because marital status and educational level are categorical variables without a linear trend, we set dummy variables for the two variables respectively. For marital status, “married” was set as the reference group. For educational level, “Junior college” was set as the reference group. In step 2, the independent variable WIF or FIW was added. In step 3, PsyCap was added. In addition, Sobel test was used to test the statistical significance of the mediation effect.

## Results

Demographic and working characteristics of subjects and distributions of each dimension of burnout in categorical items were shown in Table 1. Mean emotional exhaustion, mean cynicism and mean professional efficacy differed across age groups. Mean professional efficacy differed across marital status groups. Mean emotional exhaustion and mean cynicism differed between income groups. And mean emotional exhaustion differed between working hour groups.

**Table 1:** Demographic and working characteristics of subjects and the distributions of dimensions of burnout in categorical items

**Variable**	**N(%)**	**Emotional exhaustion Mean (SD)**	**Cynicism Mean (SD)**	**Professional efficacy Mean (SD)**
Age (yr)		P=0.003	P=0.001	P=0.012
≤30	525(39.4%)	14.27(7.52)	8.19(6.65)	23.98(8.79)
31-39	441(33.1%)	14.06(7.32)	8.33(6.21)	24.11(8.69)
≥40	366(27.5%)	12.57(8.04)	6.76(6.55)	25.67(9.35)
Education		P=0.645	P=0.594	P=0.741
High school or under	216(16.2%)	13.44(7.80)	7.42(6.58)	24.82(8.55)
Junior college	714(53.6%)	13.64(7.81)	7.88(6.66)	24.65(9.07)
Undergraduate or above	402(30.2%)	14.00(7.05)	7.98(6.11)	24.29(8.72)
Marital Status		P=0.873	P=0.600	P=0.005
Single	333(25.0%)	13.91(7.42)	8.17(6.53)	23.28(8.54)
Married/Cohabitation	967(72.6%)	13.70(7.61)	7.78(6.45)	25.09(8.91)
Divorced/Widow/Separated	32(2.4%)	13.35(7.65)	7.39(7.32)	25.84(8.45)
Monthly income		P<0.001	P<0.001	P=0.166
≤3000RMB(≈470 dollars)	823(61.8%)	14.49(7.69)	8.42(6.67)	24.19(9.00)
>3000RMB	509(38.2%)	12.57(7.31)	7.05(6.16)	24.89(8.79)
Weekly working hours		P<0.001	P=0.092	P=0.989
≤40 hours	709(53.2%)	12.80(7.40)	7.56(6.54)	24.52(9.16)
>40 hours	623(46.8%)	15.23(7.69)	8.21(6.46)	24.51(8.60)

### Correlations among work-family conflict, PsyCap and burnout

Results of Pearson correlation were shown in Table 2. Both WIF and FIW were significantly correlated with dimensions of burnout. WIF and FIW were positively related with emotional exhaustion and cynicism respectively. However, the effects of WIF and FIW on professional efficacy dimension were different. While FIW had a detrimental impact on professional efficacy, WIF depicted a positive relationship with professional efficacy among nurses. Both WIF and FIW were negatively correlated with PsyCap (Table 2).

**Table 2:** Means, standard deviations (SD) and correlations of continuous variables

**Variables**	**Mean**	**SD**	**1**	**2**	**3**	**4**	**5**	**6**
1.Age	34.53	8.55						
2.Emotional Exhaustion	13.71	7.62	-0.114**					
3.Cynicism	7.83	6.50	-0.116**	0.694**				
4.Professional Efficacy	24.49	8.93	0.073**	0.121**	-0.044			
5.WIF	3.41	0.74	0.101**	0.481**	0.342**	0.062*		
6.FIW	2.54	0.81	-0.116**	0.214**	0.354**	-0.206**	-0.438**	
7.PsyCap	4.15	0.61	0.127**	-0.270**	-0.345**	0.328**	-0.103**	-0.158**

### The mediating role of PsyCap in the relationship between work-family and emotional exhaustion

As shown in Table 3, WIF was positively associated with emotional exhaustion (*β* = 0.467, P < 0.001) whereas PsyCap was negatively associated with it (*β* = -0.205, P < 0.001). PsyCap had a partially mediating effect in the relationship between WIF and emotional exhaustion as the regression coefficient for WIF shrank when PsyCap was added (from *β* = 0.467 to *β* = 0.451; Sobel test, z = 3.50,P < 0.001).

**Table 3:** Hierarchical linear regression analysis results of WIF

**Variable**	**Emotional exhaustion**			**Cynicism**			**Professional efficacy**		
	**step 1(β)**	**step 2(β)**	**step 3(β)**	**step 1(β)**	**step 2(β)**	**step 3(β)**	**step 1(β)**	**step 2(β)**	**step 3(β)**
Age	-0.058	-0.034	-0.006	-0.092*	-0.074*	-0.030	0.034	0.036	-0.012
Dummy_m1	-0.041	-0.016	-0.006	-0.020	-0.002	0.014	-0.054	-0.052	-0.068
Dummy_m2	-0.020	-0.018	-0.019	-0.015	-0.014	-0.016	0.012	0.012	0.015
Dummy_e1	-0.023	0.004	-0.009	-0.014	0.006	-0.015	-0.006	-0.004	0.019
Dummy_e2	0.008	-0.059*	-0.058	0.010	-0.038	-0.037	-0.008	-0.015	-0.016
Monthly income	-0.105**	-0.076**	-0.065	-0.059	-0.039	-0.022	0.041	0.044	0.026
Weekly working hours	0.140**	0.093**	0.074	0.020	-0.014	-0.044	0.004	-0.001	0.030
WIF		0.467**	0.451**		0.339**	0.314**		0.050*	0.077**
PsyCap			-0.205**			-0.315**			0.341**
R^2^	0.040	0.247	0.287	0.0116	0.124	0.219	0.010	0.012	0.124
Δ R^2^	0.040**	0.207**	0.040**	0.016*	0.108**	0.095**	0.010**	0.002*	0.112**

Note: Dummy_m1 means “Single” vs. “Married/Cohabitation”, Dummy_m2 means “Divorced/Widow/Separated” vs. “Married/Cohabitation”; Dummy_e1 means “High school or under” vs. “Junior College”, Dummy_e2 means “Undergraduate or above” vs. “Junior College”.

*P<0.05 **P<0.01 (two-tailed).

As shown in Table 4, FIW was also positively associated with emotional exhaustion (β = 0.200, P < 0.001) whereas PsyCap was negatively associated with it (β = -0.216, P < 0.001). PsyCap had a partially mediating effect in the relationship between FIW and emotional exhaustion as the regression coefficient for FIW diminished when PsyCap was added (from β = 0.200 to β = 0.170; Sobel test, z = 4.91, P < 0.001).

**Table 4:** Hierarchical linear regression analysis results of FIW

**Variable**	**Emotional exhaustion**			**Cynicism**			**Professional efficacy**		
	**step 1(β)**	**step 2(β)**	**step 3(β)**	**step 1(β)**	**step 2(β)**	**step 3(β)**	**step 1(β)**	**step 2(β)**	**step 3(β)**
Age	-0.058	-0.044	-0.015	-0.092*	-0.066	-.026	0.034	0.019	-0.023
Dummy_m1	-0.041	-0.048	-0.035	-0.020	-0.032	-.015	-0.054	-0.047	-0.065
Dummy_m2	-0.020	-0.012	-0.015	-0.015	-0.002	-.006	0.012	0.004	0.009
Dummy_e1	-0.023	-0.013	-0.028	-0.014	0.003	-.018	-0.006	-0.016	0.006
Dummy_e2	0.008	0.007	0.005	0.010	0.008	.006	-0.008	-0.007	-0.005
Monthly income	-0.105**	-0.107**	-0.094**	-0.059	-0.063	-.046	0.041	0.043	0.025
Weekly working hours	0.140**	0.132**	0.111**	0.020	0.006	-.023	0.004	0.011	0.041
FIW		0.200**	0.170**		0.350**	0.310**		-0.201**	-0.158**
PsyCap			-0.216**			-0.296**			0.313**
R^2^	0.040	0.079	0.123	0.016	0.136	0.219	0.010	0.049	0.142
Δ R^2^	0.040**	0.039**	0.044**	0.016*	0.120**	0.083**	0.010**	0.040*	0.093**

Note: Dummy_m1 means “Single” vs. “Married/Cohabitation”, Dummy_m2 means “Divorced/Widow/Separated” vs. “Married/Cohabitation”; Dummy_e1 means “High school or under” vs. “Junior College”, Dummy_e2 means “Undergraduate or above” vs.”Junior College”.

*P<0.05 **P<0.01 (two-tailed).

### The mediating role of PsyCap in the relationship between work-family conflict and cynicism

As shown in Table 3, WIF was positively associated with cynicism (β = 0.339, P < 0.001) whereas PsyCap was negatively associated with it (β = -0.315, P < 0.001). PsyCap had a partially mediating effect in the relationship between WIF and cynicism as the regression coefficient for WIF shrank when PsyCap was added (from β = 0.339 to β = 0.314; Sobel test, z = 3.63, P < 0.001).

As shown in Table 4, FIW was also positively associated with cynicism (β = 0.350, P < 0.001) whereas PsyCap was negatively associated with it (β = -0.296, P < 0.001). PsyCap had a partially mediating effect in the relationship between FIW and cynicism as the regression coefficient for FIW diminished when PsyCap was added (from β = 0.350 to β = 0.310; Sobel test, z = 5.24, P < 0.001).

### The mediating role of PsyCap in the relationship between work-family conflict and professional efficacy

As shown in Table 3, WIF was positively associated with professional efficacy (β = 0.050, P < 0.05) whereas PsyCap was also positively associated with it (β = 0.341, P < 0.001). PsyCap did not have a mediating role in the relationship between WIF and professional efficacy as the regression coefficient for the WIF did not diminish when PsyCap was added.

As shown in Table 4, FIW was negatively associated with professional efficacy (β = -0.201, P < 0.001) whereas PsyCap was positively associated with it (β = 0.313, P < 0.001). PsyCap had a partially mediating effect in the relationship between FIW and professional efficacy as the absolute value of regression coefficient for FIW shrank when PsyCap was added (from β = -0.201 to β = -0.158; Sobel test, z = -5.22, P < 0.001).

## Discussion

In the present study, subjects were selected from hospitals in every sized city in Liaoning Province, China. A large sample and a high effective response rate seemed to be able to provide a good representation of our study population and enhance the generalization of our study conclusion.

Regarding the relationship between work-family conflict and burnout, WIF and FIW were found to be positively related to emotional exhaustion and cynicism, respectively, among Chinese female nurses. These results were correspondent with the results of previous studies [10,32]. Yavas et al [10] found that WIF and FIW could lead to emotionally exhausted and Fuss et al [32] indicated that high values of WIF were significantly correlated to higher rates of personal burnout. However, the effects of WIF and FIW on professional efficacy were different. While WIF had a positive relationship with professional efficacy, FIW had a negative relationship with it. One explanation of the positive relationship between WIF and professional efficacy might be that employees who experienced interpersonal conflict and tension at work tended to focus more on their work activities to protect themselves from further tension and to be able to reach higher levels of performance, which may result in higher levels of professional efficacy. This similar positive relationships was also observed in investigations of van Dyne et al [33] and Yavas [10]. From another perspective, the different relationships of WIF and FIW with professional efficacy might be explained that nurses who experienced more WIF devoted more time and energy to work, whereas the dimension of professional efficacy mainly self-evaluated based on work roles rather than family roles, thus nurses who devoted more to work might achieve more professional efficacy. By contrast, nurses who experienced more FIW devoted more time and energy to family, at the expense of interfering work-related obligations. This might deprive nurses of opportunities to achieve more work accomplishment.

These findings should urge hospital administrators to be aware of the risk of work-family conflict. Efforts should be made to develop strategies to decrease nurses’ perceived WIF and FIW to reduce nurses’ burnout. For example, hospital administrators could increase staff and give nurses opportunities to enhance their technical skills to better fit in a high-tech environment to reduce WIF. Or hospitals may provide child-care services for nurses with young kids or arrange flexible schedules for breastfeeding nurses to release them from FIW.

As an identified positive resource to fight against stress and turnover, the effect of PsyCap on burnout was rarely investigated in previous studies. In the present study, PsyCap was found to be negatively associated with emotional exhaustion and cynicism, respectively, and positively associated with professional efficacy, among Chinese nurses. These findings contributed to the understanding that PsyCap had a main effect on burnout and was a positive resource for combating burnout among nurses.

Our findings revealed that PsyCap partially mediated the effects of WIF on emotional exhaustion and cynicism, and PsyCap also partially mediated the effects of FIW on emotional exhaustion, cynicism and professional efficacy. Nurses who perceived more WIF would be more likely to experience lower levels of PsyCap which in turn increased the possibilities of developing emotional exhaustion and cynicism. And nurses who perceived more FIW would be more likely to experience lower levels of PsyCap which in turn lead to higher levels of emotional exhaustion, higher levels of cynicism and lower levels of professional efficacy. Compared to decreasing nurses’ work-family conflict, it is a more positive and feasible strategy for hospitals to develop programs increasing PsyCap of nurses, thus to enhance the organizational performance and improve the quality of health care in a long run.

Nowadays, financial capital received lots of attentions by hospital managers in China. Hospital managers care more about advanced medical technologies than the PsyCap of their health care staff to improve organizational performance [34]. According to Luthans and colleagues, PsyCap is ‘state-like’ and is open to be developed and managed [13]. Interventions designed to enhance components of PsyCap including self-efficacy, hope, resilience and optimism have been introduced in previous studies and confirmed to be effective [17,18,35]. Therefore, strategies of enhancing nurses’ PsyCap should be developed in China as soon as possible in future days.

Several limitations of the present study have to be mentioned. First, this study is the cross-sectional design. Work-family conflict, PsyCap and burnout were measured simultaneously, so causal conclusions cannot be drawn. All findings obtained in the current study should be confirmed by a longitudinal study. Second, we conducted this study in large general hospitals given that nurses in these hospitals were more likely to experience burnout, compared to nurses in community health centers. Therefore, we initially focused our attention on nurses from large general hospitals, and nurses from community health centers will be investigated in further researches. Third, all data were self-reported, which can introduce bias. Participants may have underestimated or overestimated the relationship between work-family conflict and burnout. Last but not the least, this study only focused on the relationship between work-family conflict and burnout and only checked some basic socio-demographic and working variables as confounders. More risk factors and possible confounders should be investigated in further studies.

## Conclusions

To summarize, our findings revealed that both WIF and FIW of nurses were positively related with emotional exhaustion and cynicism, respectively, among Chinese nurses. The effects of WIF and FIW on professional efficacy were different. While WIF depicted a positive relationship with professional efficacy, FIW had a detrimental impact on professional efficacy. PsyCap mediated the effects of WIF on emotional exhaustion and cynicism, and also mediated the effects of FIW on emotional exhaustion, cynicism and professional efficacy. Hence, interventions to decrease Chinese nurses’ work-family conflict and to enhance their PsyCap should be developed in future days.

## Competing interests

The authors declare that they have no conflict of interest.

## Authors’ contributions

YW was involved in all aspects of the paper including design of the study, questionnaire survey, analysis and interpretation of data, drafting and revising the manuscript and approval of the final version. YC and JLF were both involved in questionnaire survey and draft of the manuscript. LW made substantive intellectual contributions to the interpretation of data and draft of the manuscript. All authors have read and approved the final manuscript.

## Pre-publication history

The pre-publication history for this paper can be accessed here:

http://www.biomedcentral.com/1471-2458/12/915/prepub
